# Generation of a monoclonal antibody recognizing the heavily glycosylated CD45 protein and its application on identifying circulating tumor cells

**DOI:** 10.1371/journal.pone.0192506

**Published:** 2018-02-09

**Authors:** Weikai Zhang, Zhitao Li, Zihua Wang, Chunyan Yue, Hui Zheng, Ren Li, Mingxing Zhou, Zhiyuan Hu, Zewen Wei, Qin Li

**Affiliations:** 1 Medical College, Henan University of Science and Technology, Luoyang, P. R. China; 2 Department of Biomedical Engineering, School of Life Science, Beijing Institute of Technology, Beijing, P. R. China; 3 CAS Key Laboratory for Biomedical Effects of Nanomaterials and Nanosafety, CAS Center for Excellence in Nanoscience, National Center for Nanoscience and Technology of China, Beijing, P. R. China; 4 Sino-Danish College, University of Chinese Academy of Sciences, Beijing, P. R. China; 5 Yangtze River Delta Academy of Nanotechnology and Industry Development Research, Jiaxing, Zhejiang Province, P. R. China; The Ohio State University, UNITED STATES

## Abstract

Here, we provide direct evidence that using recombinant proteins expressed in eukaryotic cells as antigen is a practical way to generate monoclonal antibodies (mAbs) against heavily glycosylated proteins. Heavily glycosylated proteins are typically difficult targets for mAb generation, being limited by unsatisfactory affinity and low specificity. Using the heavily glycosylated CD45 protein as an example, we demonstrate the entire process of expressing the protein in eukaryotic cells and using it as an antigen to generate CD45-targeting mAbs in mice. The mAbs generated showed robust affinity and specificity, which are crucial factors for differentiate circulating tumor cells from white blood cells in human breast cancer patient samples. Only 1 cell fusion and 2 cyclic sub-cloning steps were necessary before mAbs with satisfactory performance were obtained.

## Introduction

Purchasing commercial mAbs is commonly done in biological laboratories to avoid the process of preparing mAbs, which is considered time- and labor-intensive [1, 2]. However, the antigen used to prepare commercial mAbs is, in most cases, not the same protein used in the customers' studies. Therefore, fluctuations in specificity between commercial mAbs and the targeted protein are inevitable [2, 3]. Moreover, economic considerations have prompted most commercial mAb providers to employ prokaryotic proteins, or peptides, instead of eukaryotically expressed proteins as the antigen. The lack of post-translational modification (for instance, glycosylation) can compromise the consistency and repeatability of studies involving glycosylated proteins [4]. To avoid these flaws of commercial mAbs, generating mAbs in one’s own laboratory may be considered as a feasible alternative, especially while performing studies on post-translationally modified proteins. For example, immunofluorescent labeling of the common leukocyte antigen, CD45, has become the most commonly used method to differentiate leukocytes from circulating tumor cells (CTCs) [5–7]. However, the CD45 expressed on the surface of leukocytes from patients is post-translationally modified heavily with N- and O-linked glycosylation [8]. Possible mismatches between native CD45 and commercial antibodies may markedly lower the reliability of CTC-enumeration results, which is a key issue for CTC-related clinical practices.

In this study, we demonstrated the generation of a mAb recognizing the heavily glycosylated human CD45 protein and its application oappn identifying CTCs. Instead of utilizing a prokaryotic protein or peptide, we used the eukaryotic protein as the antigen. Antibody preparation, including mouse immunization, hybridoma screening, and mAb characterization was completed in 3 months, as only 1 time hybridoma fusion and 2 cyclic sub-cloning steps were performed to acquire the anti-CD45 mAb, which showed greater affinity and consistency than commercial mAbs. This study provides direct evidence that using eukaryotic proteins to generate mAbs could become a practical solution for biological laboratories encountering affinity and specificity issues with commercial mAbs. The efforts of plasmid construction and eukaryotic protein preparation paid off in terms of less time consumption and enhanced binding affinity.

## Methods and materials

### Materials

A mouse anti-his tag mAb (B1169), a commercial Alexa647-conjugated anti-CD45 mAb (BDLS0479), an Alexa647 mouse IgG1 isotype control (BDLS0031), rProtein G Beads (BDTL0003-1) and a mouse IgG control (mIgG, BF01006) were obtained from Beijing Biodragon Immunotechnologies Co., Ltd. A horseradish peroxidase (HRP)-conjugated goat anti-mouse IgG antibody (M21001S) was purchased from Abmart Shanghai Co., Ltd. Complete Freund's adjuvant (F5881), incomplete Freund's adjuvant (F5506), PEG 1500 (807489) were purchased from Sigma–Aldrich (USA). Fetal bovine serum (FBS; 10099–141), Phosphate-buffered saline (PBS), Roswell Park Memorial Institute (RPMI) 1640 medium (SH30809.01B) and Dulbecco's modified Eagle's medium (DMEM; SH30243.01B) were obtained from Thermo Scientific, USA.

To express the recombinant human CD45-his (rhCD45-his) protein, human embryonic kidney HEK-293T cells were cultured in DMEM supplemented with 10% FBS, and 50 μg/mL penicillin and streptomycin. HL60 (human promyelocytic leukemia cells), SKBR-3 (human breast cancer cells), and Sp2/0-Ag14 (a mouse myeloma cells) cells were obtained from National Infrastructure of Cell Line Resource (China) and cultured in RPMI 1640 medium supplemented with 10% FBS, 50 μg/mL penicillin, and streptomycin. All cells were incubated at 37°C in a 5% CO_2_ humidified atmosphere.

### Animal ethics

We obtained the necessary licenses and approvals from the Institutional Animal Care and Use Committee of Peking University (Permit Number: SYXK [Beijing] 2016–0028), who approved all of our animal-related experiments and protocols. BALB/C mice (females weighing 20–25 g) were purchased from Vital River Laboratory Animal Technology (China). Animals were maintained in a sterile environment and feed sterilized food. At the end of the experiments, the animals were euthanized by carbon dioxide.

### Research Ethics

We had received ethical approval from the Ethics Committee of Beijing Cancer Hospital. Informed consent was provided by 5 patients (who donated blood samples) and was approved by Institutional Review Board and Beijing Cancer Hospital and signed by 5 patients. All procedures were performed in accordance with relevant guidelines and regulations. Vacutainer tubes containing the anticoagulant ethylene-diaminetetraacetic acid were used to collect blood samples (4 mL from each individual). The samples were placed at room temperature and processed within 24 h.

### Glycosylated protein expression, purification, and examination

cDNA encoding human CD45 was amplified by reverse transcriptase-PCR from total RNA extracted from HL60 cells and then cloned into the pMD19-T vector. Using the pMD19-T vector (3721, Takara Biomedical Technology), as the template, the fragment encoding extracellular CD45 was amplified and inserted into the pCDNA3.1 vector (CV003, Sino Biological Inc.) to generate the recombinant vector pCDNA3.1-CD45-his. cDNA encoding the CD45 ectodomain was amplified with the primers:

5′-GGAATTGTACCCGCGGGCCCCGCCACCATGCAAAGCCCAACACCTTCCC-3′ (forward)

and 5′ -GATCTCGGTCGACCGAATTCTCAATGATGATGATGATGATGCTTAGAATTATAAG ATGTTG-3′ (reverse).

The inserted nucleotide sequence was verified by DNA sequencing. The recombinant vector pCDNA3.1-CD45-his was transfected into HEK293T cells using Lipofectamine 2000. The recombinant protein was purified using Ni-NTA agarose (Beijing Biodragon Immunotechnologies, China). The protein purity was analyzed by sodium dodecyl sulfate-polyacrylamide gel electrophoresis (SDS-PAGE) and its concentration was measured using the BCA Assay Kit (Solarbio, China). Finally, 2 mg protein was obtained from 200 mL cell culture supernatants and stored at –20°C.

For glycosylation analysis, the rhCD45-his protein was reduced for 40 min with 5 mM dithiothreitol in 25 mM NH_4_HCO_3_ at room temperature and alkylated for 40 min with 15 mM iodoacetamide in 25 mM NH_4_HCO_3_ in the dark. After being washed and dehydrated, the alkylated sample was digested overnight at 37°C with trypsin (Promega, USA), using a 1: 50 enzyme: substrate ratio. After digestion, the peptide mixtures were extracted with 50% acetonitrile and 0.1% trifluoroacetic acid for 2 h at 37°C. The peptide sample was dried, dissolved in 10 μL 0.1% formic acid, and subjected to nano liquid chromatography-tandem mass spectrometry (LC-MS/MS) analysis.

For western blot assays, the rhCD45-his protein was reduced with loading buffer and boiled for 5 min. Two micrograms rhCD45-his protein and 2 μg PD-L1-his protein were added to separate lanes. After electrophoresis and blotting, the blots were blocked in PBS containing 5% skim milk and 0.1% Tween-20 (mPBST) at room temperature and then incubated with a primary antibody (mouse anti-his monoclonal antibody) in mPBST overnight at 4°C. The blots were washed in PBS with 0.1% Tween-20 (PBST) and incubated with an HRP-conjugated goat anti-mouse IgG secondary antibody in mPBST for 1 h at room temperature. The blots were washed again before the bands were detected.

### Mice immunization and hybridoma screening

For the first mouse immunization, a 500-μL sample (50 μg rhCD45-his protein dissolved in 250 μL PBS plus 250 μL complete Freund’s adjuvant) was subcutaneously injected into each mouse. For the following 2 immunizations, complete Freund’s adjuvant was replaced by incomplete Freund’s adjuvant. The interval between each immunization was 2 weeks.

The mouse which exhibited the highest antiserum titer was sacrificed to retrieve its splenocytes. For the cell fusion, approximately 1.2 x 10^8^ splenocytes were mixed with 2.4 x 10^7^ myeloma Sp2/0-Ag14 cells, and 1 mL 50% (v/v) PEG 1500 was added to the mixture to promote fusion. The mixture was evenly split into four 96-well plates. Thus, approximately 3 x 10^7^ splenocytes were seeded into each plate. All cells were cultured for 5 days in RPMI medium with HAT reagent to exclude unfused cells. Each well contained at least 1 hybridoma clone.

Enzyme-linked immunosorbent assay (ELISA) and flow cytometry were used to screen for hybridomas that secreted anti-CD45 antibodies. Hybridoma cell lines were established after 2 cycles of sub-cloning. ELISA plates were coated with rhCD45-his protein (1 μg/mL) or betaine homocysteine s-methyltransferase (BHMT)-his protein (1μg/mL) in carbonate buffer and incubated overnight at 4°C. Then, the plates were blocked with PBS containing 5% milk (mPBS) and incubated for 1 h at 4°C. The primary antibody consisted of antiserum collected from immunized mice (or the purified CD45 antibody) diluted in mPBS, which was incubated with the ELISA plate for 1 h at 37°C. After being washed 3 times with PBST, the plates were incubated for 1 h at 37°C with an HRP-conjugated goat anti-mouse IgG (1: 5000), which was diluted in mPBST. Tetramethylbenzidine was used as substrate to catalyze HRP reactions for 10 min, and 50 μL 2M H_2_SO_4_ was used to stop the reaction. The absorbance signal was measured at 450 nm.

### mAb characterization

To purify antibodies, ascites were diluted with 10-fold PBS and injected to Protein G affinity chromatography (BDTL0004-5, Beijing Biodragon Immunotechnologie). Alexa647 dye was conjugated to antibody 4D3 by employing a Protein Labeling Kit (A20173, Invitrogen detection technologies).

For surface plasmon resonance (SPR) assays, 2 μL rhCD45 protein (1 μg/μL) was immobilized on a SPR chip (Nanocapture Gold, Plexera LLC, USA) surface modified with a gold-layer and incubated overnight at 4°C. The chip was blocked in 5% skim milk overnight at 4°C, washed 3 times in PBS, and dried under a stream of nitrogen. The Alexa647-4D3 and Alexa647-CmAb were diluted in PBST at different concentrations (133, 66.5, 33.3, 16.6, 8.31, and 4.17 nM). SPR analysis was performed with the following cycle: running buffer (PBST, for baseline stabilization); sample (from low to high concentrations) for the binding assays (association phase); running buffer (PBST as dissociation phase, for washing), and 0.5% (v/v) H3PO4 in deionized water (for chip regeneration). After 1 cycle was finished, the next sample was started on the same chip until all samples were completed. Real-time binding signals were recorded and analyzed using the PlexArray HT system (Plexera LLC, USA). The dissociation constant was calculated by fitting the data to association–dissociation curves.

For immunofluorescence analysis, cells were harvested, washed twice with PBS, and fixed with 4% paraformaldehyde. Cells were incubated with 4',6-diamidino-2-phenylindole (DAPI) dye (1:100) for 10 min at room temperature to stain the cell nuclei, after which they were centrifuged for 3 min at 2000 rpm before the supernatant was removed. Five microliters of an Alexa647-labeled antibody (25 μg/mL) was added. After a 1-h incubation at room temperature, the cells were washed in PBS and fluorescently imaged.

For flow cytometry assays, HL60 cells were collected and washed twice in PBS. Cells were incubated with 100uL culture supernatant or 5 μL of Alexa647-labeled anti-CD45 (25 μg/mL) for 1 h at room temperature, while mIgG1 was used as a negative control. In addition, lymphocytes were separated from blood using Lymphocyte Separation Medium (TBD Science, China).

### Enumerating CTCs

Two milliliters of peripheral blood from cancer patients was diluted with 5 mL PBS in a 15-mL tube and incubated directly with immunomagnetic beads (Bio-Adembeads Streptavidin 0312, ADEMTECH Inc) modified with a peptide targeting the epithelial cell adhesion molecule (EpCAM) for 1 h at room temperature [9]. EpCAM-positive cells were enriched with a magnetic shelf. Thirty minutes later, the supernatant was removed, and the enriched cells were re-suspended in 5 mL PBS. The process of enriching EpCAM-positive cells was repeated 3 times. Two hundred microliters of paraformaldehyde (4%) was added to the tube for 15 min to fix the enriched cells, followed by cell staining with DAPI for 15 min. All cells were treated with 400 μL 0.3 M glycine solution containing 10% normal goat serum for 1 h, and then 20 μL each of Alexa647-labelled anti-CD45 and fluorescein isothiocyanate (FITC)-labeled anti-CK19 antibodies were used to label cells for 1.5 h. Subsequently, the cells were re-suspended in PBS and transferred from the centrifugal tube to a glass slide, on which the cells were fluorescently imaged and identified. All procedures were performed at room temperature.

## Results

**Fig 1:**
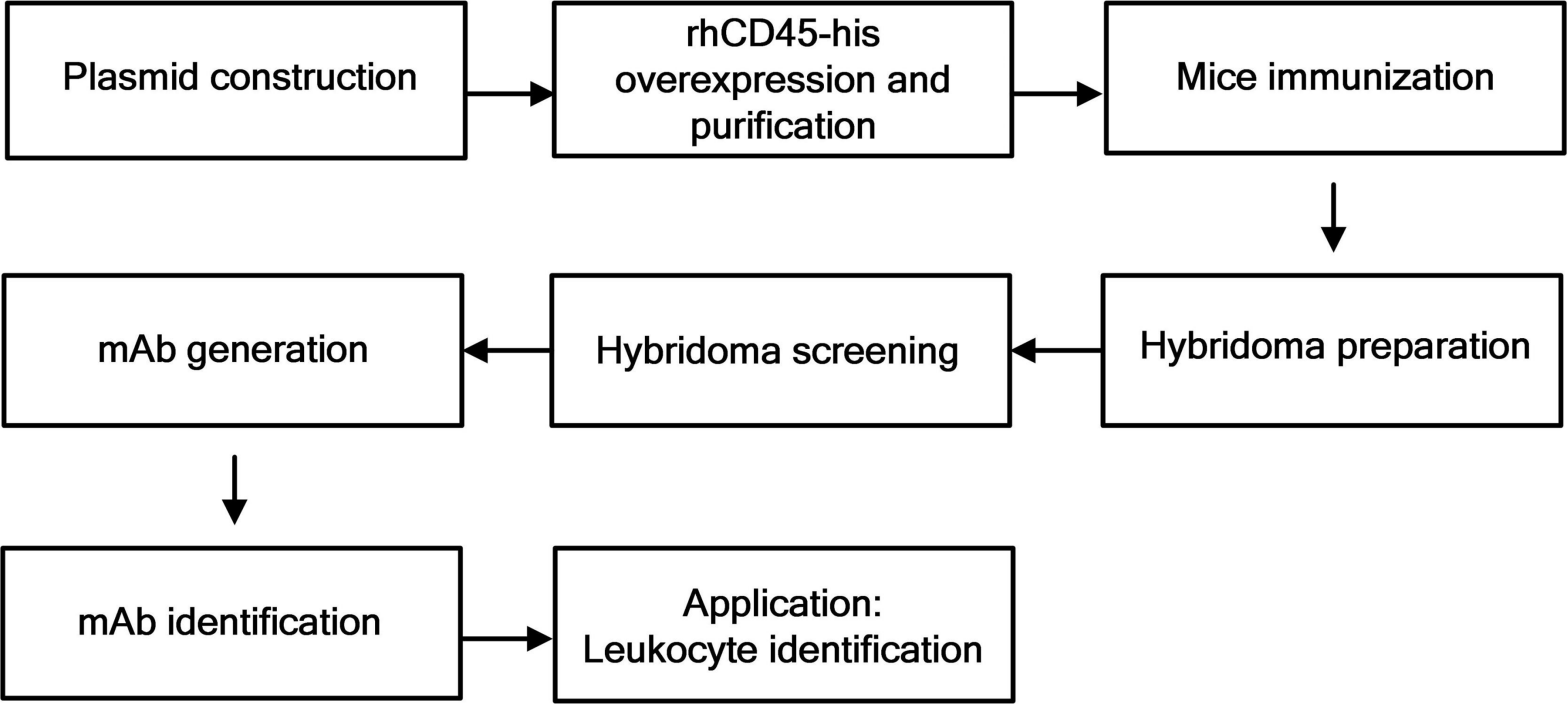
Schematic representation of the experimental procedures used in this study. The experimental procedures consists of 4 parts: expressing the glycosylated rhCD45-his protein, generating CD45-targeting antibodies, screening mAbs, and applying it on CTC enumeration.

The overall experimental procedure used in this study is represented schematically in Fig 1. Briefly, a eukaryotic expression plasmid was constructed and transfected into HEK293T cells to produce the glycosylated recombinant human CD45-his protein. After collection and purification, the expressed glycosylated rhCD45-his protein was used to immunize mice. When the antiserum titer was determined to be satisfactory, the mice were sacrificed to retrieve their splenocytes, which were fused with Sp2/0-Ag14 cells to produce hybridomas. The positive hybridoma clones were collected and characterized to generate mAbs, which were further used in immunofluorescent assays to identify leukocytes.

### Expression of the glycosylated rhCD45-his protein

**Fig 2:**
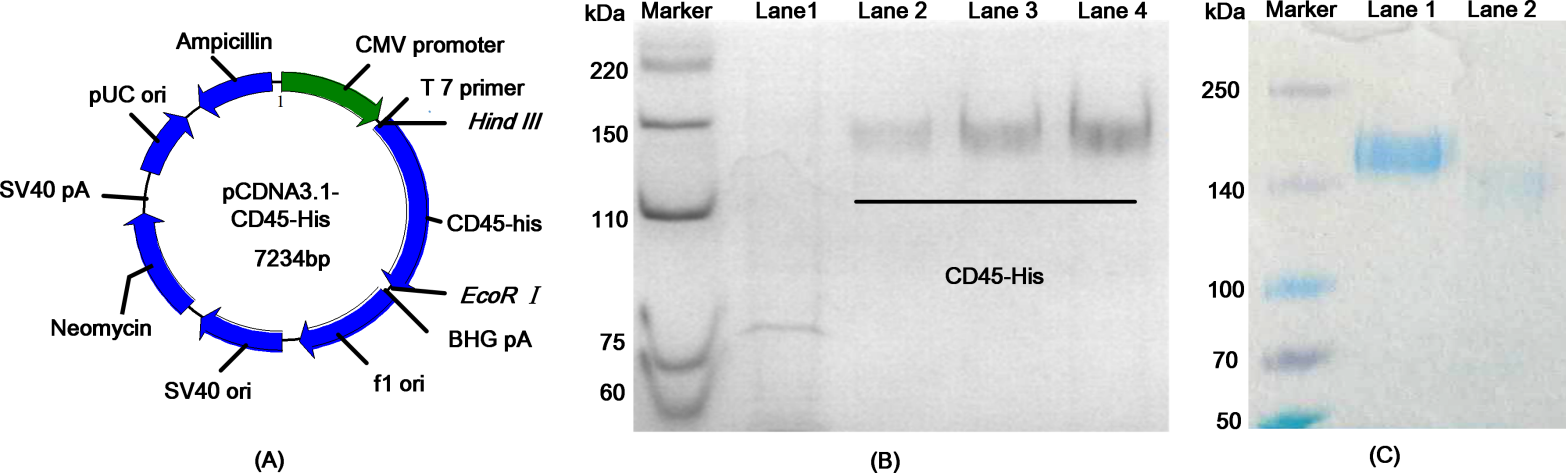
Expression and identification of the glycosylated rhCD-45 protein. (A) Construction of the recombinant vector pCDNA3.1-CD45-his. A gene encoding the CD45 ectodomain with a C-terminal 6x his-tag was inserted into the pCDNA3.1 vector. (B) The expressed rhCD45-his protein was analyzed by SDS-PAGE. Lane 1: supernatant from HEK293T cells; lanes 2–4: 1, 2, and 4 μg purified recombinant protein. The recombinant protein was purified from the supernatant by Ni-NTA agarose. (C) A reduced SDS-PAGE with PNGase F treatment was used to ensure the existence of sugars. Compared with un-treated protein (lane 1), the PNGase F treatment (lane 2) reduces the molecular weight by eliminating N-glycan.

CD45 is a type-I transmembrane glycoprotein expressed abundantly on the surfaces of all leukocytes [10]. The native CD45 protein expressed on the surface of leukocytes has extensive post-translational modification in the form of N-linked and O-linked glycosylation [10, 11]. To produce CD45 in heavily glycosylated form, we generated the eukaryotic expression plasmid pCDNA3.1-CD45-his, encoding a 5′ signal peptide, the extracellular domain of CD45, and a 3′ 6× his tag (Fig 2A). Accurate construction of the pCDNA3.1-CD45-his plasmid was verified by sequencing (as shown in S1 Fig). The recombinant vector was transfected into HEK293T cells using Lipofectamine 2000. At 72 h post-transfection, the rhCD45-his protein was purified from the culture supernatant by immobilized affinity chromatography with nickel-nitrilotriacetic acid (Ni-NTA) agarose. As shown in Fig 2B, SDS-PAGE analysis demonstrated that the CD45-his protein was successfully purified. The predicted molecular mass of the CD45 protein is 60 kDa. The protein gel of 6% reduced SDS-PAGE revealed that the molecular mass of rhCD45-his was approximately 140 kDa, suggesting that extensive post-translational modifications occurred. An anti-his mAb was further used for western blotting to detect CD45-his. As shows in S2 Fig, glycosylated CD45 was successfully expressed in HEK293T cells. As shown in Fig 2C, a reduced SDS-PAGE with PNGase F treatment was used to ensure the existence of sugars. Compared with un-treated protein (lane 1), the PNGase F treatment (lane 2) reduces the molecular weight by eliminating N-glycan.

We then used LC-MS/MS to further investigate the post-translational modifications in detail. 39 glycopeptides were detected on 12 amino acids (as shown in S3 Fig), hinting that the same amino acid was modified by various glycans.

### Mice immunization and hybridoma screening

**Fig 3:**
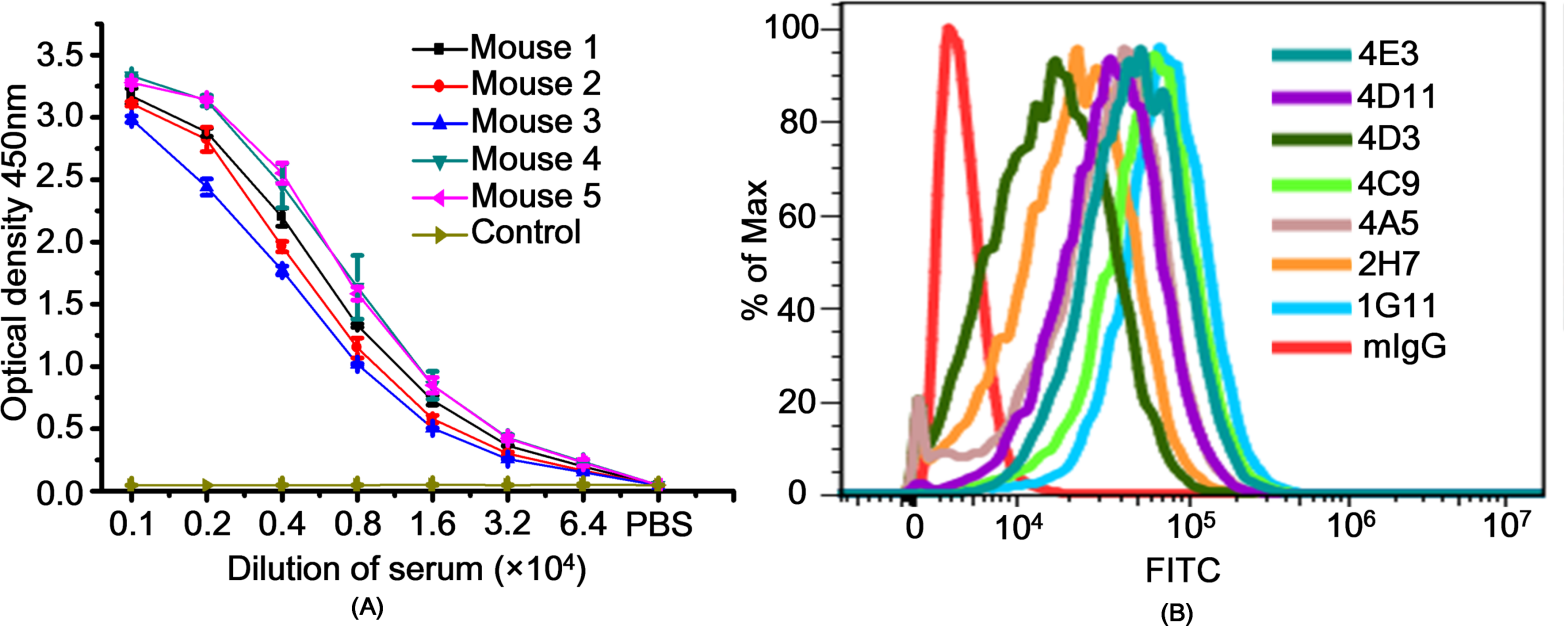
Antiserum titer analysis and hybridoma screening. (A) Antiserum titers against the rhCD45-his protein were determined from the tail blood samples of 5 mice. Mouse IgG and blood from a non-immunized mouse were used as negative controls. The rhCD45-his protein (1μg/mL) was coated on an ELISA plate, and the plate was blocked with PBS containing 5% milk (mPBS). Then antisera were serially 2-fold diluted (from 1: 0.1 × 10^4^ to 1: 6.4 × 10^4^) and added to the ELISA plate. An HRP-conjugated goat anti-mouse IgG was used as the secondary antibody. The cutoff value was set as 2.1 times the control value. Data are presented as the mean ± SD of measurements derived from 2 independent assays. (B) Flow cytometry was performed to determine the binding between HL60 cells and antibodies in the hybridoma supernatants. The ratios of cell numbers were normalized to their maximum values.

After acquiring a sufficient amount of heavily glycosylated rhCD45-his protein, 5 mice were immunized 3 times at 14-day intervals. For each immunization, 500 μL (1 μg/μL) rhCD45-his protein was injected per mouse. Seven days after the second immunization, the antiserum titers were determined by indirect ELISA. As shown in Fig 3A, the antibody titers of all mice were more than 1: 32,000, which was high enough for hybridoma preparation and screening [12]. Using normal mouse serum as the negative control and PBS as the blank control, we employed indirect ELISA to investigate the reaction between the rhCD45-his protein and supernatant from each well. Supernatants from 25 wells exhibited reactions with the rhCD45-his protein (as shown in S4 Fig). Considering that his-tag could also induce immune responses, we used the irrelevant his-tagged protein, BHMT-his [13], to perform another indirect ELISA assay and exclude possible antibody recognition of his-tag. As shown in S5 Fig none of the 25 screened supernatants reacted with the BHMT-his protein. Although the rhCD45-his protein expressed in eukaryotic cells was glycosylated, its spatial conformation could be different than that of the native protein expressed on leukocyte membranes. Thus, we performed flow cytometry to investigate the reaction between the antibodies in the 25 screened supernatants and HL60 acute promyelocytic leukemia cells, which express glycosylated CD45 on the cell membrane. Compared with mouse IgG, which was used as a negative control, antibodies in 7 supernatants showed clear binding to HL60 cells (Fig 3B). After performing routine sub-cloning twice to exclude unstable hybridomas, we finally obtained 4 positive hybridomas.

Table 1 summarizes several key parameters of the hybridoma-screening procedures. Starting from the first mouse immunization, the entire immunization and hybridoma-screening process required only 2 months to acquire 4 positive hybridomas. Only 1 cell fusion and 2 cycles of sub-cloning were performed. In contrast, in previous studies [4] employing peptides or prokaryotic protein as the antigen, 2 or more cell fusions and hybridoma screens were performed. For most laboratories conducting biological research, efficient time and labor management are key factors influencing the decision whether to generate specific mAbs in house.

**Table 1:** Hybridoma preparation and screening.

Number of wells	Wells containing clones	Supernatant analysis		Screened hybridomas
		ELISA	FACS	
**372**	**100% (372/372)**	**6.72% (25/372)**	**1.8% (7/372)**	**4**

For all 372 wells, at least 1 hybridoma clone was observed in each well. After screening hybridomas by an indirect ELISA (detecting binding of the purified rhCD45-his protein) and flow cytometry (detecting binding with native CD45 on the cell membrane), 4 hybridomas were further characterized.

### mAb purification and characterization

**Fig 4:**
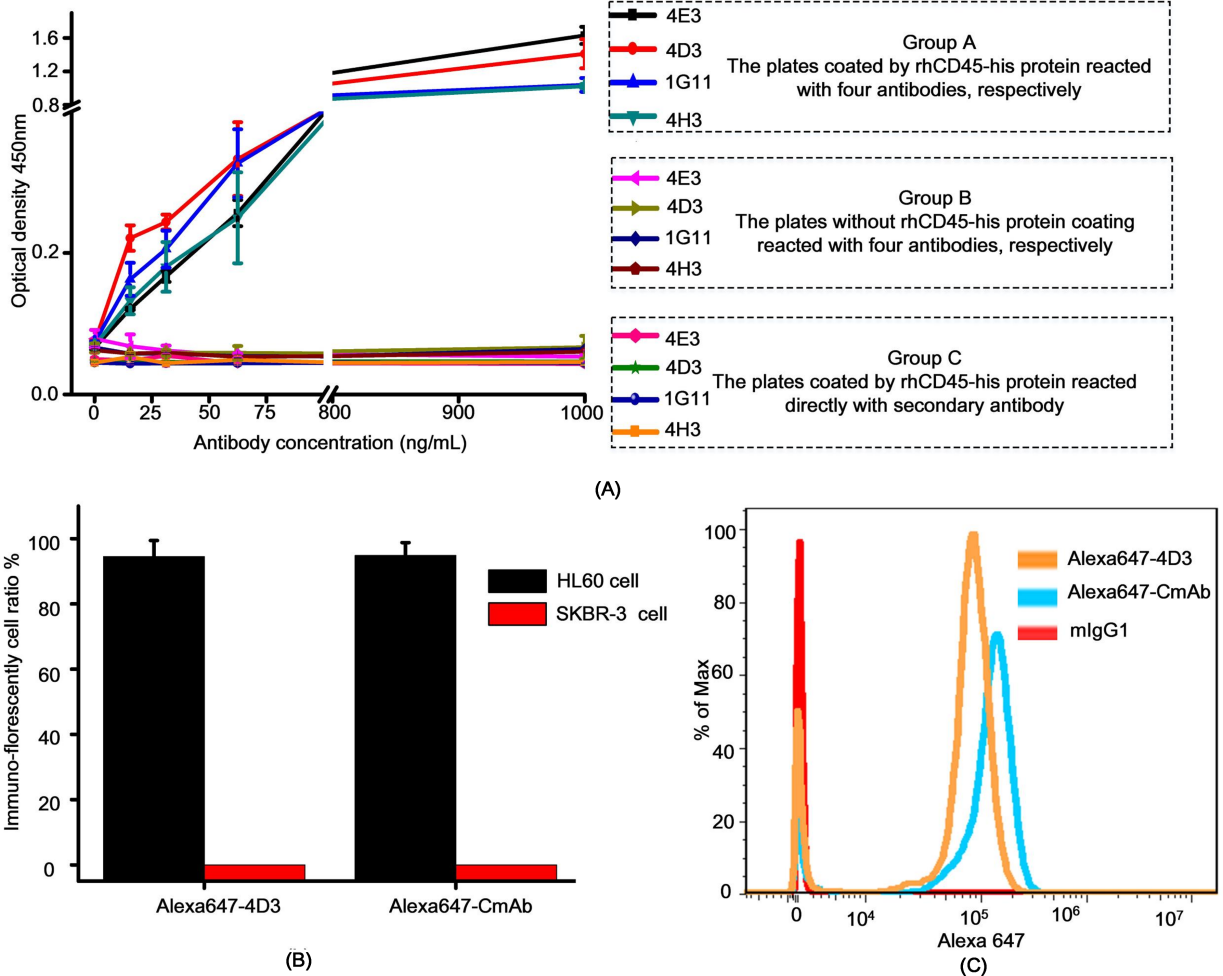
Monoclonal antibody screening and characterization. The screened antibody 4D3 was fluorescently labeled with the dye Alexa647 and designated as Alexa647-4D3, and a commercial antibody was designated as Alexa647-CmAb. (A) Determining the affinities of antibodies in recognizing the rhCD45-his protein. Group A: The rhCD45-his protein (1 μg/mL) was coated on the ELISA plates for overnight at 4°C, and the plates were blocked with mPBS at 37°C. Four antibodies (designated as 4E3, 4D3, 1G11 and 4H3) were serially diluted 2-fold from 1000 ng/mL to 15.6 ng/mL and added to separate wells. An HRP-conjugated goat anti-mouse IgG antibody was used as the secondary antibody. Group B: Control group, no protein was coated on ELISA plate. Group C: Control group, no antibody 4D3 was added. For all groups, the optical density cutoff value was set as 0.2. Data are presented as the mean ± SD of measurements derived from 3 independently assays. (B) Evaluating the specificity between antibodies and CD45-positive cells (HL60) and CD45-negative cells (SKBR-3 cell). Cells were stained with Alexa647-4D3 and Alexa647-CmAb, as indicated. The percentage of stained HL60 cells was calculated from the fluorescent images, using the NIH-recommended, Image J software. Data are presented as the mean ± SD of 3 independent assays. (C) Flow cytometry was used to evaluate the binding between antibodies and CD45-positive lymphocytes. Lymphocytes were isolated from 2 mL patient blood, divided evenly into 3 parts, and separately stained with Alexa647-CmAb, Alexa647-4D3, and an Alexa647-labeled mouse IgG1 antibody, which was used as a negative control. The fractions of positive cell were normalized to the maximum value.

After establishing 4 hybridoma cell lines, which secreted antibodies that strongly reacted with the glycosylated CD45 protein, 1 x 10^6^ hybridoma cells were injected intraperitoneally into a mouse that was pre-treated with liquid paraffin. After 7 days, ascites were collected, and antibodies were purified by protein G affinity chromatography. Then, the antibody subtype was determined. As shown in S6 Fig, all antibodies were of the IgG1 subtype. Antibody affinities were analyzed by indirect ELISA (Fig 4A) with proper controls. The 4D3 mAb had the highest affinity. In addition, SDS-PAGE and size exclusion chromatography (SEC) were employed to determine antibody purities (S7C and S7A Fig). The results revealed that the purity of 4D3 was 97.7% (S7B Fig). Therefore, mAb 4D3 was selected for subsequent assays. Limiting dilution was used to sorting single cell clones, and this method is theoretically capable of ensuring monoclonality. To further experimentally confirm that 4D3 is monoclonal, the accurate molecular weight of 4D3 antibody was analyzed by LC-MS (S8 Fig). The result exhibits 27 clear and independent peaks, without any irrelevant noise signal, demonstrating that monoclonal antibody is acquired.

**Fig 5:**
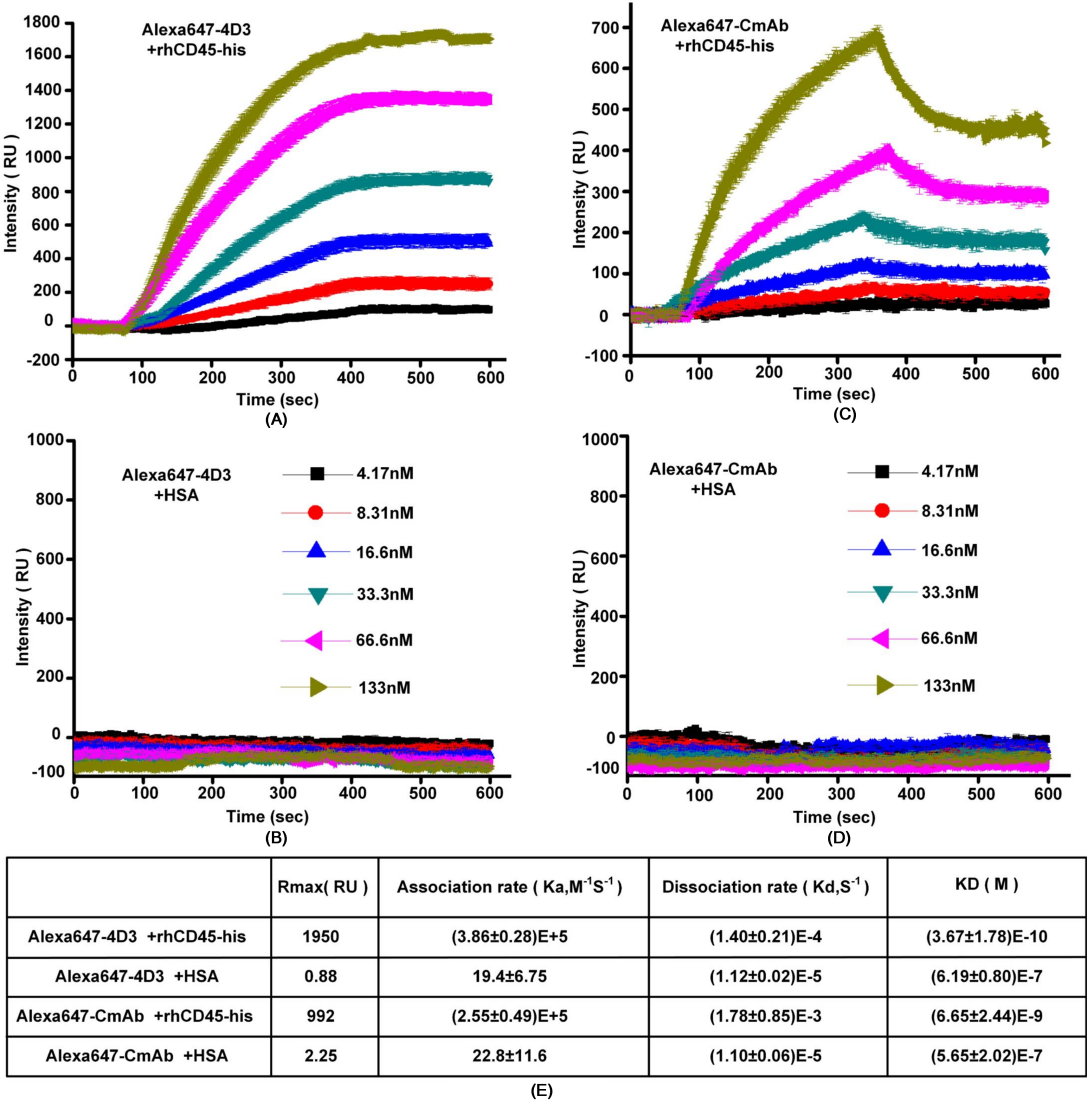
SPR assays of screened mAb 4D3 and a commercial mAb. The affinities of Alexa647-4D3 (A) and Alexa647-CmAb (C) were determined by SPR. The rhCD45-his protein (1 mg/mL) was immobilized on the SPR plate, and antibodies were respectively added at different concentrations (133.3, 66.5, 33.3, 16.7, 8.33, and 4.17 nM). Association and dissociation curves were recorded. Another two HSA protein coated SPR plates were used as controls for Alexa647-4D3 (B) and Alexa647-CmAb (D), respectively. (E), Parameters were calculated from the curves of 3 times measurements.

The purpose of producing an anti-CD45 mAb was to immunofluorescently detect glycosylated CD45 on leukocyte membranes. Thus, we fluorescently labeled mAb 4D3 with the fluorescent dye, Alexa647, and the resulting fluorescent labeled mAb was designated Alexa647-4D3. For comparison, we also purchased a commercial anti-CD45 mAb labeled with Alexa647, which was named as Alexa647-CmAb. To quantitatively investigate the binding affinity between Alexa647-4D3 and the glycosylated CD45 protein, we performed SPR assays to calculate the KD, which is widely accepted when characterizing antibody–antigen binding affinities [14]. Compared with Alexa647-CmAb (Fig 5C), Alexa647-4D3 (Fig 5A) had a lower KD. However, the calculated data exhibited considerable errors. Therefore, it was considered that Alexa647-4D3 had similar affinity with Alexa647-CmAb. To further evaluate the binding between mAbs and native glycosylated CD45 protein on cell surface, we performed immunofluorescence assays with HL60 cells (which express CD45 on the cell membrane) and SKBR3 cells (which are CD45-negative). By directly counting the number of immunofluorescently labeled HL60 cells, it was found that Alexa647-4D3 and Alexa647-CmAb showed similar performance (Fig 4B). Apart from the affinity, the specificity was also evaluated by performing immunofluorescence assays with the HL60 and SKBR3 cells. Both Alexa647-4D3 and Alexa647-CmAb bound to HL60 cells, but not SKBR3 cells (S9 Fig).

### Clinical application of identifying CTCs

**Fig 6:**
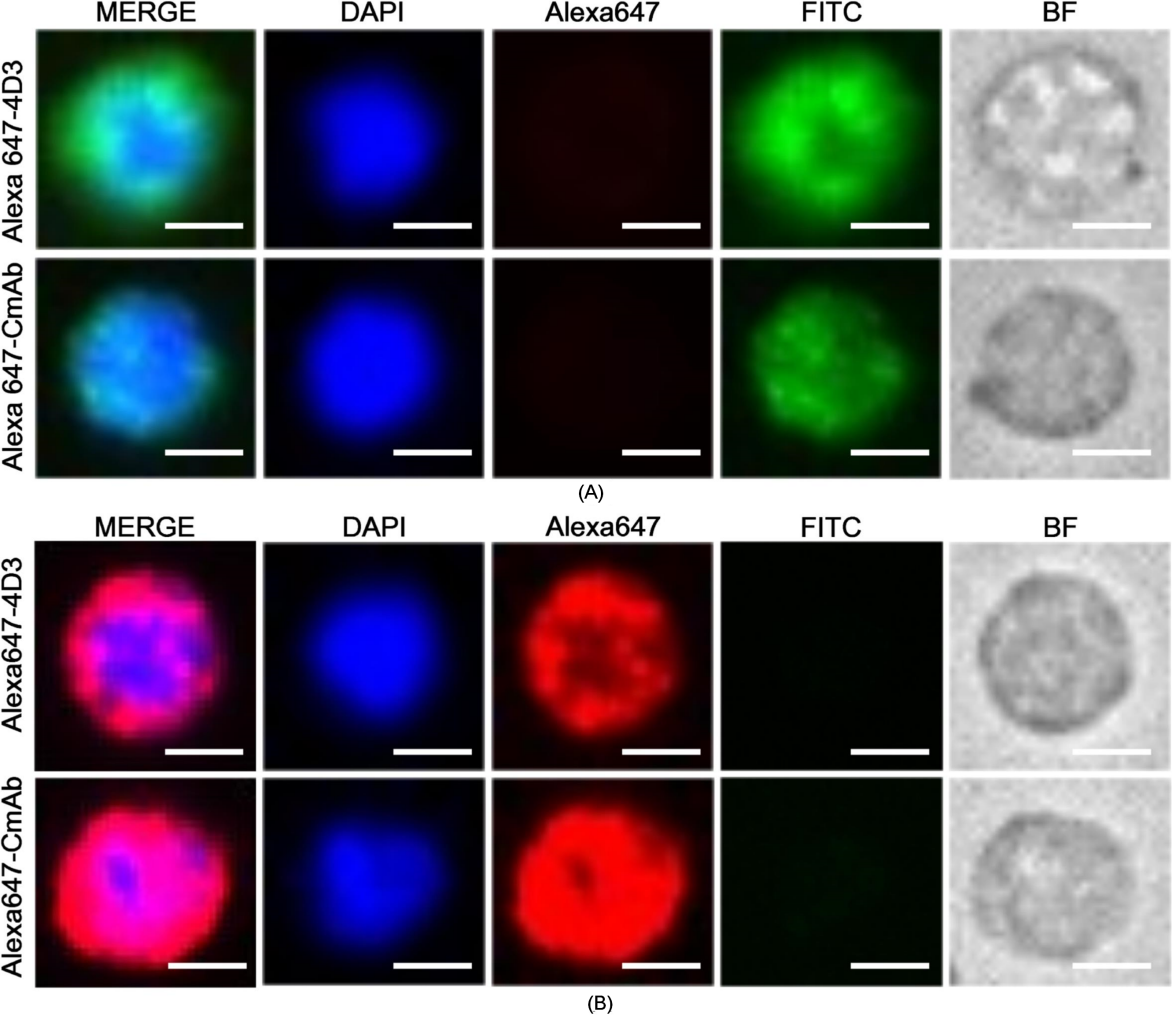
CTC identification. For CTC enumeration, EpCAM-positive cells were first isolated from patient blood samples, after which they were immunofluorescently identified. DAPI (blue) was used to stain the cell nucleus. Alexa-647-labeled anti-CD45 antibodies (red) were used to stain the leukocyte marker CD45. FITC-labeled anti-CK19 antibodies (green) were used to stain CK19, which is widely accepted as a marker of breast cancer CTCs. Therefore, CTCs (A) exhibited a status of DAPI+/CD45-/CK19+, which were colored blue and green, but not red. Typical leukocytes (B) were DAPI+/CD45+/CK19- and colored blue and red, but not green. Scale bar: 5 μm.

Enumerating CTCs in the blood has been demonstrated to be helpful for selecting cancer treatment [15]. Although multiple methods have proven effective in isolating CTC-like cells, definitive identification of CTCs still relies on immunofluorescent recognizing specific proteins on the cell membrane. For instance, CK19, a cytokeratin belonging to the type I group, was used to identify breast cancer cells [16, 17] whereas CD45 was used to exclude lymphocyte. We first isolated lymphocytes from patient blood samples to investigate binding between the mAbs and CD45 on lymphocyte membranes. As shown in Fig 4C, our flow cytometry results demonstrated that Alexa647-4D3 and Alexa647-CmAb performed similarly. To ensure that the Alexa647-4D3 binds all types of lymphocytes, we employed diverse cell surface markers to differentiate lymphocyte subsets, while evaluating Alexa647-4D3 binding to each subset. The results (S10 Fig) demonstrated that Alexa647-4D3 recognized all lymphocyte subsets, including T cells, B cells, and NK cells. To enable the clinical application of Alexa647-4D3, we tested mAbs with blood samples from 4 female breast cancer patients. Patient 1 was at stage 2a and receiving second-line chemotherapy; patient 2 was at stage 3b and receiving fourth-line chemotherapy; patient 3 was at stage 2b and receiving endocrine therapy; and patient 4 was at stage 2a and receiving third-line chemotherapy. As shown in Fig 6 and Table 2, Alexa647-4D3 and Alexa647-CmAb performed similarly.

**Table 2:** Enumeration of CTCs in the blood of patients with breast cancer.

	Alexa647-4D3	Alexa647-CmAb
**Patient 1**	**4 CTCs**	**3 CTCs**
**Patient 2**	**17 CTCs**	**11 CTCs**
**Patient 3**	**4 CTCs**	**5 CTCs**
**Patient 4**	**2 CTCs**	**2 CTCs**

The numbers of CTCs detected in 4 patients are shown. For each patient, a 4-mL blood sample was split into 2 equal parts, which were incubated with Alexa 647-4D3 and Alexa647-CmAb. All patients were female and had breast cancer. Patient 1 was at stage 2a and receiving second-line chemotherapy; patient 2 was at stage 3b and receiving fourth-line chemotherapy; patient 3 was at stage 2b and receiving endocrine therapy; and patient 4 was at 2a and receiving third-line chemotherapy.

Overall, Alexa647-4D3 and Alexa647-CmAb performed similarly in recognizing lymphocytes and CTC enumeration. However, before selecting Alexa647-CmAb, 2 commercial anti-CD45 mAbs which were purchased from Sino Biological (10086-MM05-F-25) and Abbiotec (251081) failed at CTC enumeration because of their weak binding with native CD45 on lymphocyte membranes (S11 Fig).

## Discussion and conclusions

A stable supply of high-quality mAbs is crucial for conducting most protein-related studies in laboratories engaged in biological research. Commercial mAbs are normally considered easy to access and helpful for saving time and labor costs (versus producing mAbs in-house). However, for unusual or heavily glycosylated proteins, purchasing commercial mAbs may not be a good choice. First, there could be a long period before shipping orders, considering that providers need to prepare antibodies from the initial immunization stage. Second and more importantly, it is likely that the specificity and affinity of commercial mAbs are uncertain, especially for specifically glycosylated proteins. For instance, fluorescent staining of heavily glycosylated CD45 on leukocytes has become the most common method for differentiating CTCs from leukocytes. The method requires large quantities of mAbs with satisfactory affinity and high specificity, as the number of CTCs are quite low (usually <100 in 2 mL blood), compared with the number of leukocyte (10^6^ in 2 mL blood). Using our own experience as an example, we spent >8 months on testing 3 kinds of antibodies from 3 different manufacturers before obtaining an acceptable mAb. However, the next batch of mAbs failed to meet our requirements. While encountering such issues, tend to simply try other antibodies or query related databases, for instance, www.antibodypedia.com. The potential impact of glycosylation on antibody recognition has not been comprehensively investigated. Still, some studies [4] inferred that the mismatch between unglycosylated antigen and glycosylated target protein might cause the fluctuations in antibody binding. The purpose of this study was to provide a reference for biological laboratories demanding appropriate mAbs and facing a similar dilemma: i.e., what kind of antigen should be used (eukaryotically expressed protein, prokaryotically expressed protein, or peptides). Although only the heavily glycosylated CD45 protein was used, this study still provides direct evidence that using a eukaryotically expressed protein as the antigen was efficient in producing mAbs targeting a heavily glycosylated protein. Efforts in plasmid construction and preparing the eukaryotic protein paid off in terms of lowering the time requirements and enhancing the binding affinity.

## Supporting information

S1 Fig Sequencing of pCDNA3.1-CD45-his. The sequencing result (lower, 81-1736bp) was consistency with data from GenBank (upper, NM_002838.4). The rhCD45-his vector was sequenced in Sangon Technology (Shanghai). The signal peptide is native.
(DOCX)

S2 Fig Western blot analysis of recombinant protein. The recombinant protein was detected by western blotting with an anti-his mAb. The protein was separated by 6% SDS-PAGE and transferred to a polyvinylidene fluoride membrane and probed with a mouse anti-his-tag mAb. His-tagged variant of the programmed cell death ligand 1 (PD-L1-his) protein was recognized with an anti-his-tag mAb, demonstrating the effectiveness of the mAb. The anti-his-tag mAb also recognized his-tag of recombinant rhCD45-his protein.
(DOCX)

S3 Fig Liquid chromatography-tandem mass spectrometry investigation of glycosylation. The experimental was performed as follow: The sample (4 μL) was loaded into reversed-phase C18-AQ resin (Maisch GmbH, Germany); Mobile phase: A: 0.1% formic acid in water; B: 0.1% formic acid in acetonitrile. Data dependent on MS/MS: up to top 5 most intense peptide ions from the preview scan in the Orbitrap. The raw MS file were analyzed and searched against the new established protein sequence database based on the theoretical sequence of the target protein using Byonic software (Version 2.3.5). Only high confident identified peptides were chosen for downstream protein modification analysis. The result indicates that 39 peptides were modified by N- and O-glycosylation on 12 amino acid sites and an amino acid site was modified with different glycans as shown in S3A Fig. The sequence of amino acid were shown in S3B Fig.
(DOCX)

S4 Fig The reaction between hybridoma supernatants and rhCD45-his protein. For culturing hybridoma, four 96-well plates were used. Among 384 wells, 372 wells were occupied by at least one hybridoma clone. The reactions between supernatant from each wells and rhCD45-his protein were determined by ELISA. For ELISA assays, the procedure is as wells as describe in method, and 12 wells were used as control, as: 4 wells were filled with antiserum from immunized mice, considered as positive control; 4 wells were filled with antiserum from unimmunized mice, and 4 wells were filled with PBS, both considered as negative control. HRP conjugated goat anti-mouse IgG was used as second antibody, adding to every well. In this figure, each colored spot represent a well. The red boxes represent 4 wells which were filled with antiserum from immunized mice. Supernatants from 25 wells were with optical densities (450nm) larger than 0.2. The related 25 hybridomas were considered positive.
(DOCX)

S5 Fig Comparison the reaction between supernatants and BHMT-his protein and CD45-his. Culture supernatants were reacted to 96 well plates coated with BHMT-his and CD45-his. As shown in S5 Fig, no supernatant was bound with BHMT-his.
(DOCX)

S6 Fig Subtype identification. Indirect ELISA was used to confirm the subtype of 4 antibodies (designated as 1G11, 4E3, 4D3, and 4H3). The rhCD45-his protein (1 μg/mL) was coated on the ELISA plate. Antibodies were purified from mice ascites and added to the ELISA plate at 1 μg/mL. HRP-conjugated goat anti-mouse IgG1, IgG2a, IgG2b, IgG3, IgA, and IgM were added to separate wells as the second antibody. Data are presented as the mean ± SD of measurements derived from 2 independent assays.
(DOCX)

S7 Fig Analyse purity of 4D3 by SEC and SDS-PAGE. Size exclusion chromatography (SEC) and (SDS-PAGE) assays have been performed to determine the purity of antibody 4D3. (A) A peak indicating the existence of antibody 4D3 was detected at 9.43 min. (B) the purity of antibody 4D3 is 97.7%. The parameters of SEC are: Flow rate: 0.7ml/min; Temperature: 25°C; 40 μL antibody 4D3 is injected into the column which is purchased from Aglilent Technologies (Agilent AdvanceBio SEC). (C) 4D3 was separated with a reduced SDS-PAGE, and two clear bands indicates heavy chain (50kDa) and light chain (20kDa). No irrelevant band was observed.
(DOCX)

S8 Fig LC-MS analysis of mAb 4D3. The accurate molecular weight of 4D3 antibody was analyzed by LC-MS. The result exhibits 27 clear and independent peaks, without any irrelevant noise signal, demonstrating that monoclonal antibody is acquired.
(DOCX)

S9 Fig Specificities of Alexa647-4D3 and Alexa647-CmAb. To evaluate the specificities of Alexa647-4D3 and Alexa647-CmAb, SKBR3 and HL60 cells were stained by Alexa647-4D3 and Alexa647-CmAb, respectively. In this figure, blue spots represent cell nucleic stained by DAPI. As expected, neither Alexa647-4D3 nor Alexa647-CmAb bound to SKBR3 cells. As to the binding with HL60 cells, Alexa647-4D3 shows similar or slightly better performance with Alexa647-CmAb. Under the same optical conditional, Alexa647-4D3 is with higher fluorescent intensity than Alexa647-CmAb. Scale bar:50 μm.
(DOCX)

S10 Fig FACS analysis of Alexa647-4D3. Flow cytometry was used to evaluate the binding between Alexa647-4D3 and lymphocyte subsets. CD3 (PECY5 labelled), CD4 (FITC labelled), CD8 (PE labelled), CD16 (PE labelled), CD19 (FITC labelled), and CD56 (FITC labelled) antibodies were introduced to differentiate lymphocyte subsets. Alexa647-4D3 was used to label all lymphocytes. S10A1, S10A2, S10B1 and S10B2 Fig demonstrated that T cells (CD3+CD4+ and CD3+CD8+) accounted for 61.83% of total lymphocyte amount while S10A3 and S10B3 Fig revealed that about 99.9% of T cells exhibited obvious binding with Alexa647-4D3. Similarly, B cells (CD3-CD19+) accounted for 14.57% of total lymphocyte amount (S10C1 and S10C2 Fig) while about 99.9% of B cells exhibited obvious binding with Alexa647-4D3 (S10C3 Fig); NK cells (CD3-CD56+CD16+) accounted for 5.1% of total lymphocyte amount (S10D1, S10D2, S10E1 and S10E2 Fig) while more than 96.4% of NK cells exhibited obvious binding with Alexa647-4D3 (S10D3 and S10E3 Fig). S10F1 and S10F2 Fig were negative controls in which no antibody was used.
(DOCX)

S11 Fig Commercial mAb targeting to CD45 antigen. Before applying commercial mAbs on CTC identification, we tested the binding between commercial mAbs and HL60 cells. One of them (Sino Biological 10086-MM05-F-25) results in no binding at all, while another one (Abbiotec 251081) is with limited binding efficiencies. (Only about 60% of HL60 cells was recognized, as shown in the S11 Fig). The binding between PE-labelled Abbiotec 251081 antibody and HL60 cells shows that about 60% HL60 cells were recognized. Scale bar: 20 μm.
(DOCX)

## References

[1] Prassas I, Brinc D, Farkona S, Leung F, Dimitromanolakis A, Chrystoja CC, et al False biomarker discovery due to reactivity of a commercial ELISA for CUZD1 with cancer antigen CA125. Clin Chem. 2014;60(2):381–388. doi: 10.1373/clinchem.2013.215236 24097894 10.1373/clinchem.2013.215236

[2] Pradidarcheep W, Labruyere WT, Dabhoiwala NF, Lamers WH. Lack of specificity of commercially available antisera: better specifications needed. J Histochem Cytochem. 2008;56(12):1099–1111. doi: 10.1369/jhc.2008.952101 18796405 10.1369/jhc.2008.952101 PMC2583905

[3] Couchman JR. Commercial antibodies: the good, bad, and really ugly. J Histochem Cytochem. 2009;57(1):7–8. doi: 10.1369/jhc.2008.952820 18854593 10.1369/jhc.2008.952820 PMC2605718

[4] Wang HW, Zheng XD, Wei HM, Tian ZG, Sun R. Preparation and functional identification of a monoclonal antibody against the recombinant soluble human NKp30 receptor. Int Immunopharmacol. 2011;11(11):1732–1739. doi: 10.1016/j.intimp.2011.06.007 21718806 10.1016/j.intimp.2011.06.007

[5] Adams DL, Stefansson S, Haudenschild C, Martin SS, Charpentier MS, Chumsri S, et al Cytometric characterization of Circulating Tumor Cells Captured by microfiltration and their correlation to the cellsearch CTC test. Cytometry A. 2015;87(2):137–144. doi: 10.1002/cyto.a.22613 25515318 10.1002/cyto.a.22613

[6] Zhang YJ, Wang F, Ning N, Chen Q, Yang Z, Guo Y, et al Patterns of circulating tumor cells identified by CEP8, CK and CD45 in pancreatic cancer. Int J Cancer. 2015;136(5):1228–1233. doi: 10.1002/ijc.29070 25042121 10.1002/ijc.29070

[7] Wu YQ, Deighan CJ, Miller BL, Balasubramanian P, Lustberg MB, Zborowski M, et al Isolation and analysis of rare cells in the blood of cancer patients using a negative depletion methodology. Methods. 2013;64(2):169–182. doi: 10.1016/j.ymeth.2013.09.006 24056212 10.1016/j.ymeth.2013.09.006 PMC3874448

[8] Trowbridge IS, Ostergaard HL, Johnson P. CD45: a leukocyte-specific member of the protein tyrosine phosphatase family. Biochim Biophys Acta. 1991;1095(1):46–56. 1834176 10.1016/0167-4889(91)90043-w

[9] Bai LL, Du YM, Peng JX, Liu Y, Wang YM, Yang YL, et al Peptide-based isolation of circulating tumor cells by magnetic nanoparticles. J Mater Chem. 2014;2(26):4080–4088. 10.1039/c4tb00456f 32261739

[10] Hermiston ML, Xu Z, Weiss A. CD45: A Critical Regulator of Signaling Thresholds in Immune Cells. Annu Rev Immunol. 2003;21(1):107–137. 12414720 10.1146/annurev.immunol.21.120601.140946

[11] Earl LA, Baum LG. CD45 Glycosylation controls T-cell life and death. Immunol Cell Biol. 2008;86(7):608–615. doi: 10.1038/icb.2008.46 18607388 10.1038/icb.2008.46

[12] Jin N, Ling SM, Yang C, Wang SH. Preparation and identification of monoclonal antibody against Citreoviridin and development of detection by Ic-ELISA. Toxicon. 2014;90:226–236. doi: 10.1016/j.toxicon.2014.08.057 25157801 10.1016/j.toxicon.2014.08.057

[13] Ma HL, Ning J, Jin X, Mao CM, Bu XL, Wang M, et al Betaine homocysteine methyltransferase (BHMT) as a specific and sensitive blood marker for acute liver injury. Biomarkers. 2014;19(7):578–584. doi: 10.3109/1354750X.2014.951880 25144858 10.3109/1354750X.2014.951880

[14] Lakayan D, Haselberg R, Niessen WM, Somsen GW, Kool J. On-line coupling of surface plasmon resonance optical sensing to size-exclusion chromatography for affinity assessment of antibody samples. J Chromatogr A. 2016;1452:81–88. doi: 10.1016/j.chroma.2016.05.033 27215465 10.1016/j.chroma.2016.05.033

[15] Satelli A, Brownlee Z, Mitra A, Meng QH, Li S. Circulating tumor cell enumeration with a combination of epithelial cell adhesion molecule- and cell-surface vimentin-based methods for monitoring breast cancer therapeutic response. Clin Chem. 2015;61(1):259–266. doi: 10.1373/clinchem.2014.228122 25336717 10.1373/clinchem.2014.228122 PMC4360893

[16] Pukazhendhi G, Glück S. Circulating tumor cells in breast cancer. J Carcinog. 2014;13(1):8. 25191136 10.4103/1477-3163.135578 PMC4141360

[17] Zhao S, Yang HK, Zhang MH, Zhang DK, Liu YP, Liu Y, et al Circulating Tumor Cells (CTCs) Detected by Triple-Marker EpCAM, CK19, and hMAM RT-PCR and Their Relation to Clinical Outcome in Metastatic Breast Cancer Patients. Cell Biochem Biophys. 2013;65(2):263–273. doi: 10.1007/s12013-012-9426-2 22990361 10.1007/s12013-012-9426-2

