# Supplementary figures and images for: Generation of a monoclonal antibody recognizing the heavily glycosylated CD45 protein and its application on identifying circulating tumor cells
Source: PLoS One. 2018 Feb 9;13(2):e0192506. doi: 10.1371/journal.pone.0192506 (PMC5806877; doi:10.1371/journal.pone.0192506)

**S1 Fig**


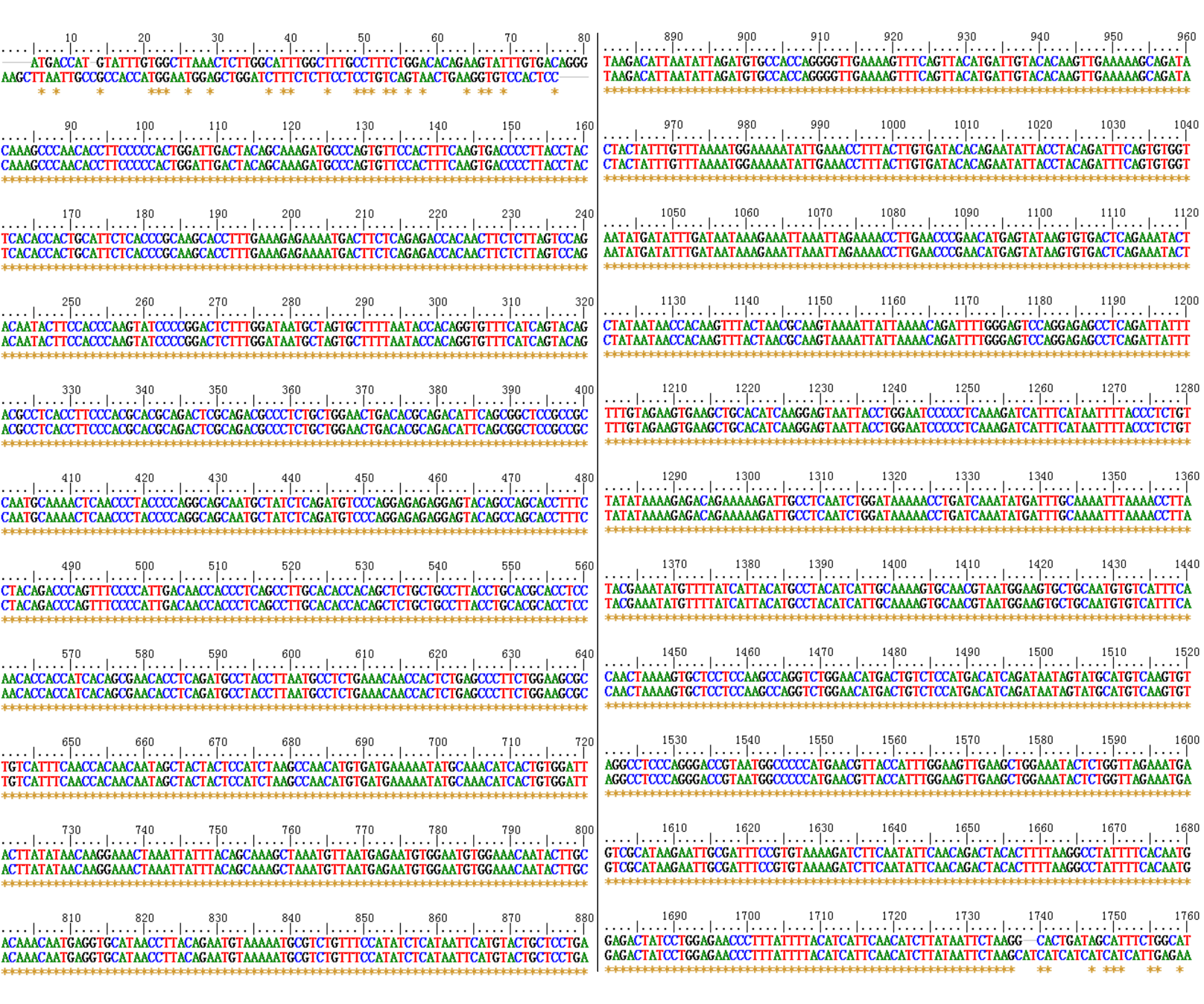

Supplement: S1 Fig — The sequencing result (lower, 81-1736bp) was consistency with data from GenBank (upper, NM_002838.4). The rhCD45-his vector was sequenced in Sangon Technology (Shanghai). The signal peptide is native. (DOCX) [file pone.0192506.s001.docx]

**S2 Fig**

**
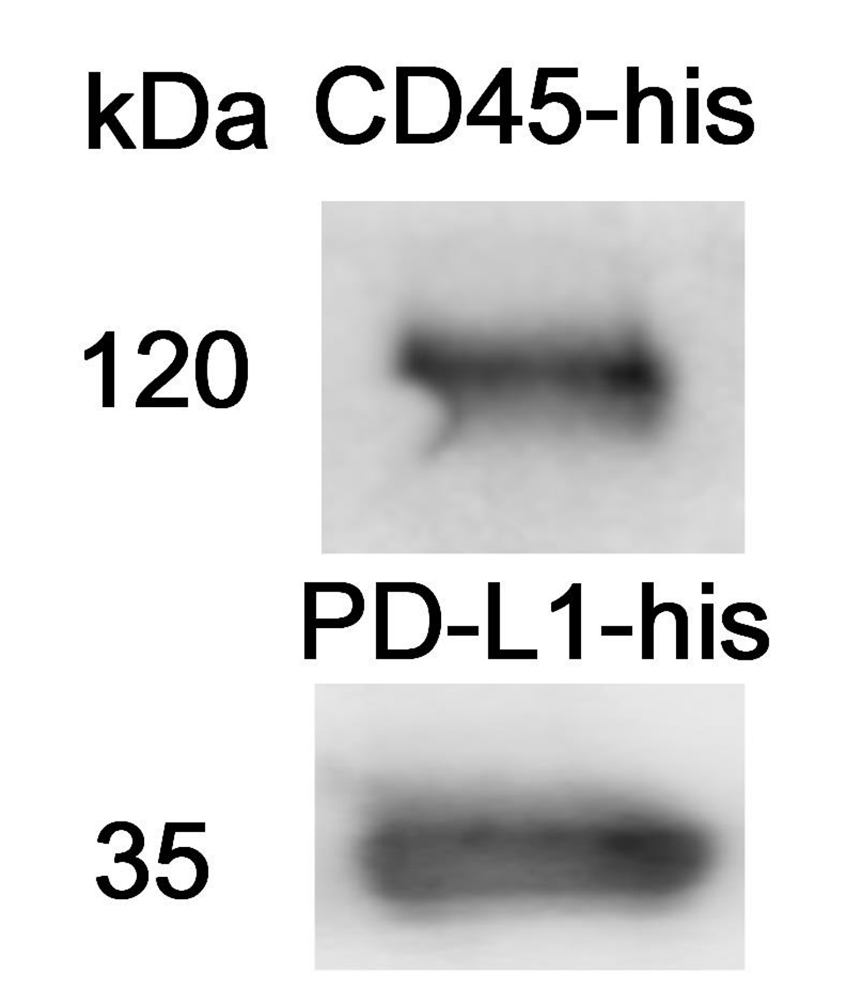
**

Supplement: S2 Fig — The recombinant protein was detected by western blotting with an anti-his mAb. The protein was separated by 6% SDS-PAGE and transferred to a polyvinylidene fluoride membrane and probed with a mouse anti-his-tag mAb. His-tagged variant of the programmed cell death ligand 1 (PD-L1-his) protein was recognized with an anti-his-tag mAb, demonstrating the effectiveness of the mAb. The anti-his-tag mAb also recognized his-tag of recombinant rhCD45-his protein. (DOCX) [file pone.0192506.s002.docx]

**S3 Fig**

**
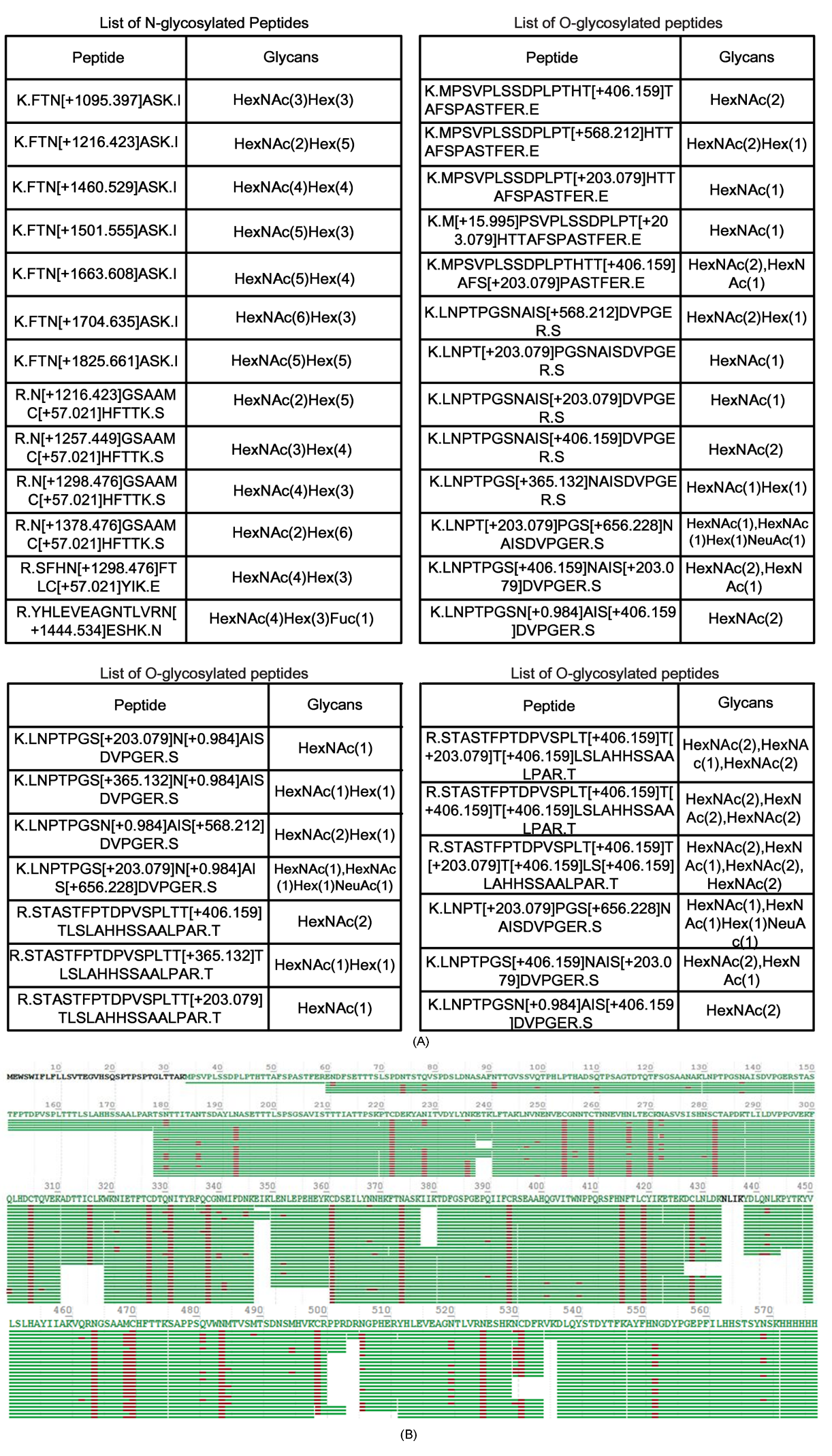
**

Supplement: S3 Fig — The experimental was performed as follow: The sample (4 μL) was loaded into reversed-phase C18-AQ resin (Maisch GmbH, Germany); Mobile phase: A: 0.1% formic acid in water; B: 0.1% formic acid in acetonitrile. Data dependent on MS/MS: up to top 5 most intense peptide ions from the preview scan in the Orbitrap. The raw MS file were analyzed and searched against the new established protein sequence database based on the theoretical sequence of the target protein using Byonic software (Version 2.3.5). Only high confident identified peptides were chosen for downstream protein modification analysis. The result indicates that 39 peptides were modified by N- and O-glycosylation on 12 amino acid sites and an amino acid site was modified with different glycans as shown in S3A Fig. The sequence of amino acid were shown in S3B Fig. (DOCX) [file pone.0192506.s003.docx]

**S4 Fig**

**
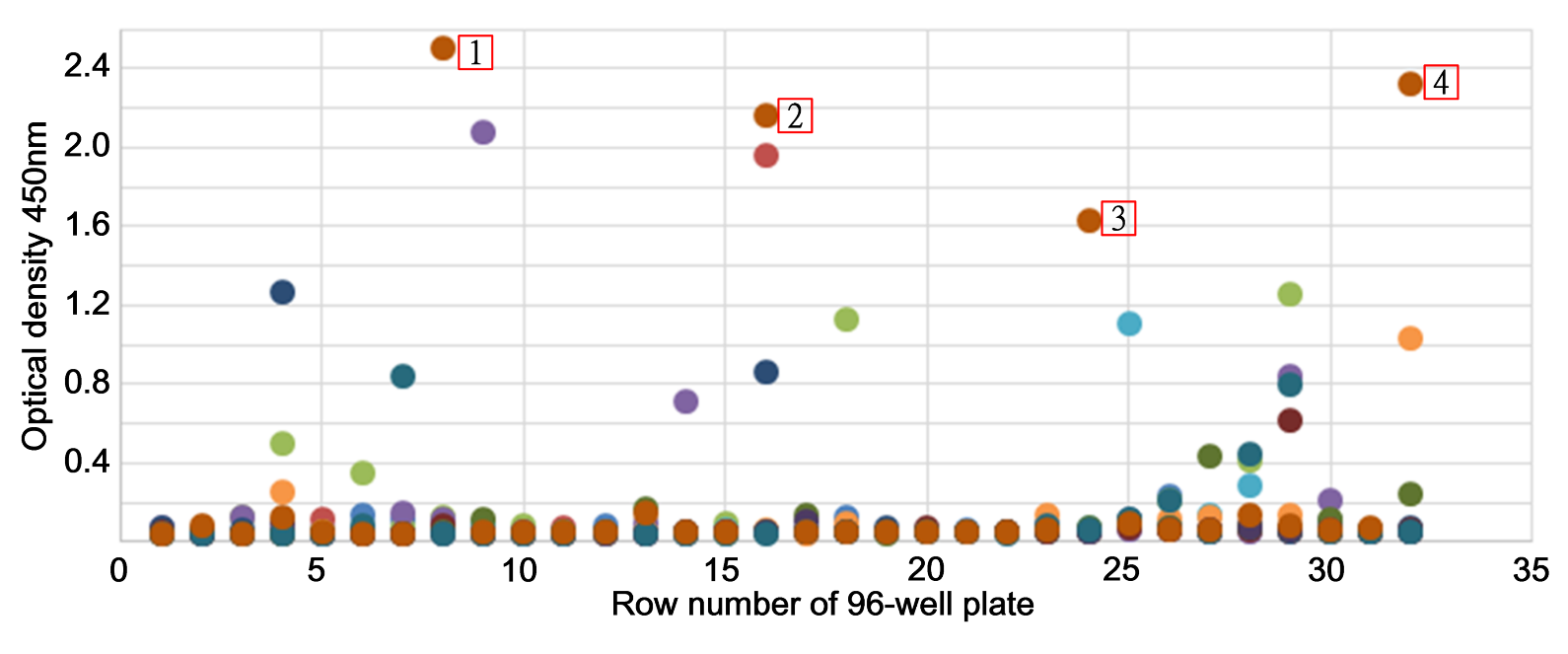
**

Supplement: S4 Fig — For culturing hybridoma, four 96-well plates were used. Among 384 wells, 372 wells were occupied by at least one hybridoma clone. The reactions between supernatant from each wells and rhCD45-his protein were determined by ELISA. For ELISA assays, the procedure is as wells as describe in method, and 12 wells were used as control, as: 4 wells were filled with antiserum from immunized mice, considered as positive control; 4 wells were filled with antiserum from unimmunized mice, and 4 wells were filled with PBS, both considered as negative control. HRP conjugated goat anti-mouse IgG was used as second antibody, adding to every well. In this figure, each colored spot represent a well. The red boxes represent 4 wells which were filled with antiserum from immunized mice. Supernatants from 25 wells were with optical densities (450nm) larger than 0.2. The related 25 hybridomas were considered positive. (DOCX) [file pone.0192506.s004.docx]

**S5 Fig**

**
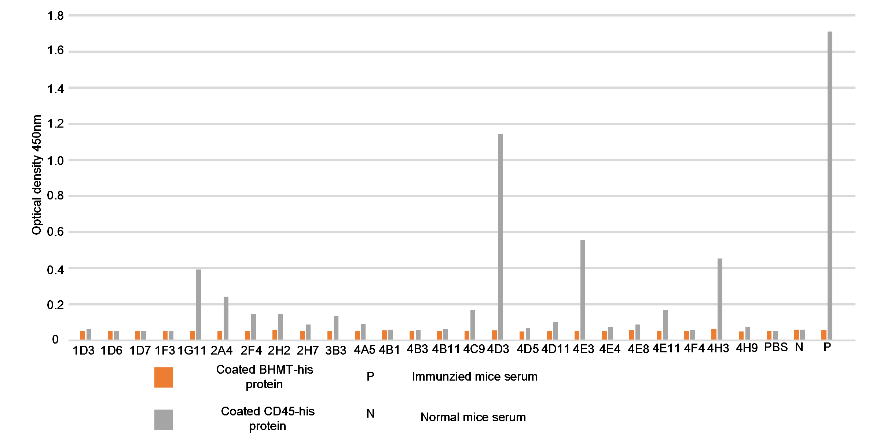
**

Supplement: S5 Fig — Culture supernatants were reacted to 96 well plates coated with BHMT-his and CD45-his. As shown in S5 Fig, no supernatant was bound with BHMT-his. (DOCX) [file pone.0192506.s005.docx]

**S6 Fig**

**
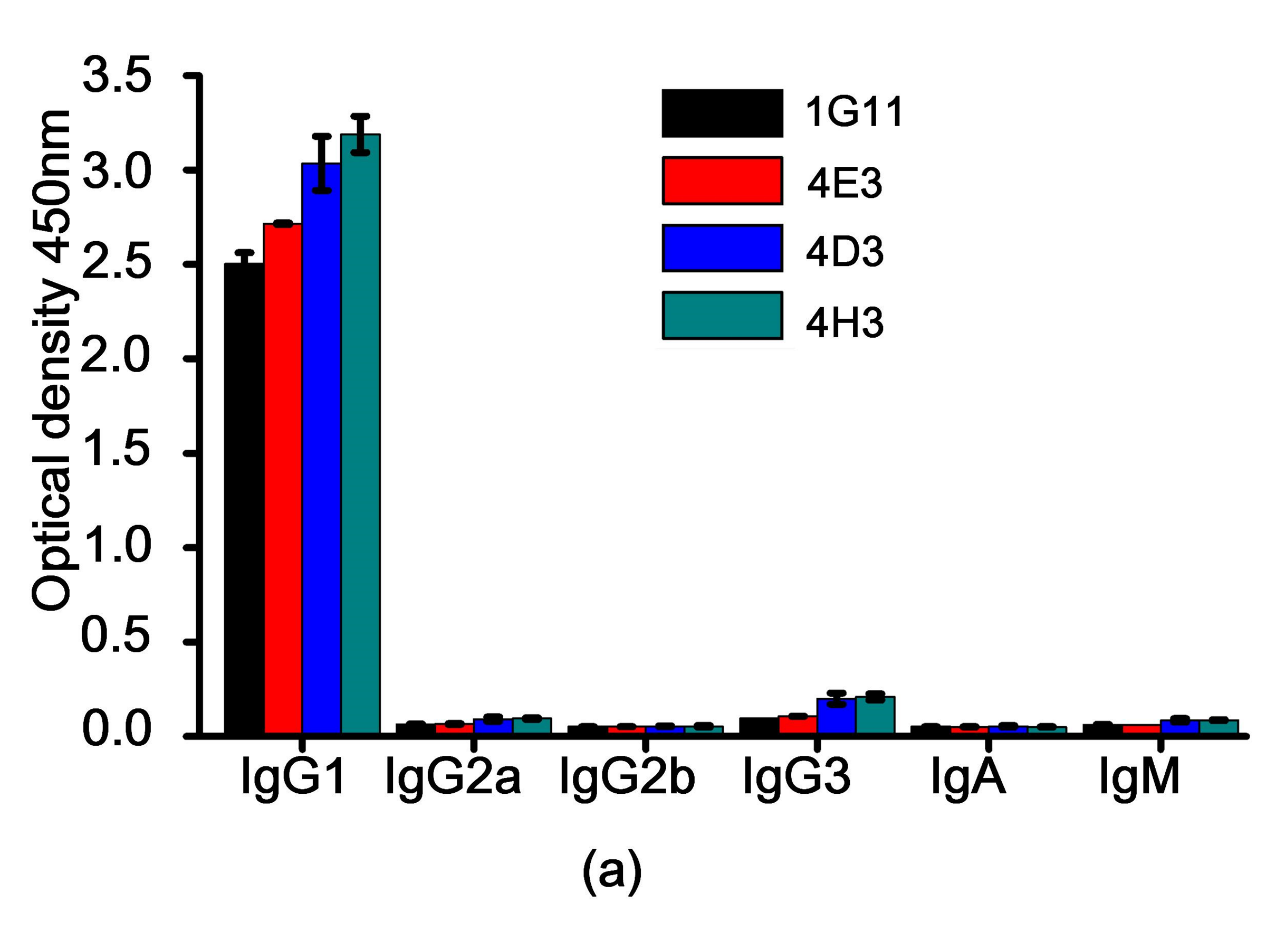
**

Supplement: S6 Fig — Indirect ELISA was used to confirm the subtype of 4 antibodies (designated as 1G11, 4E3, 4D3, and 4H3). The rhCD45-his protein (1 μg/mL) was coated on the ELISA plate. Antibodies were purified from mice ascites and added to the ELISA plate at 1 μg/mL. HRP-conjugated goat anti-mouse IgG1, IgG2a, IgG2b, IgG3, IgA, and IgM were added to separate wells as the second antibody. Data are presented as the mean ± SD of measurements derived from 2 independent assays. (DOCX) [file pone.0192506.s006.docx]

**S7 Fig**

**
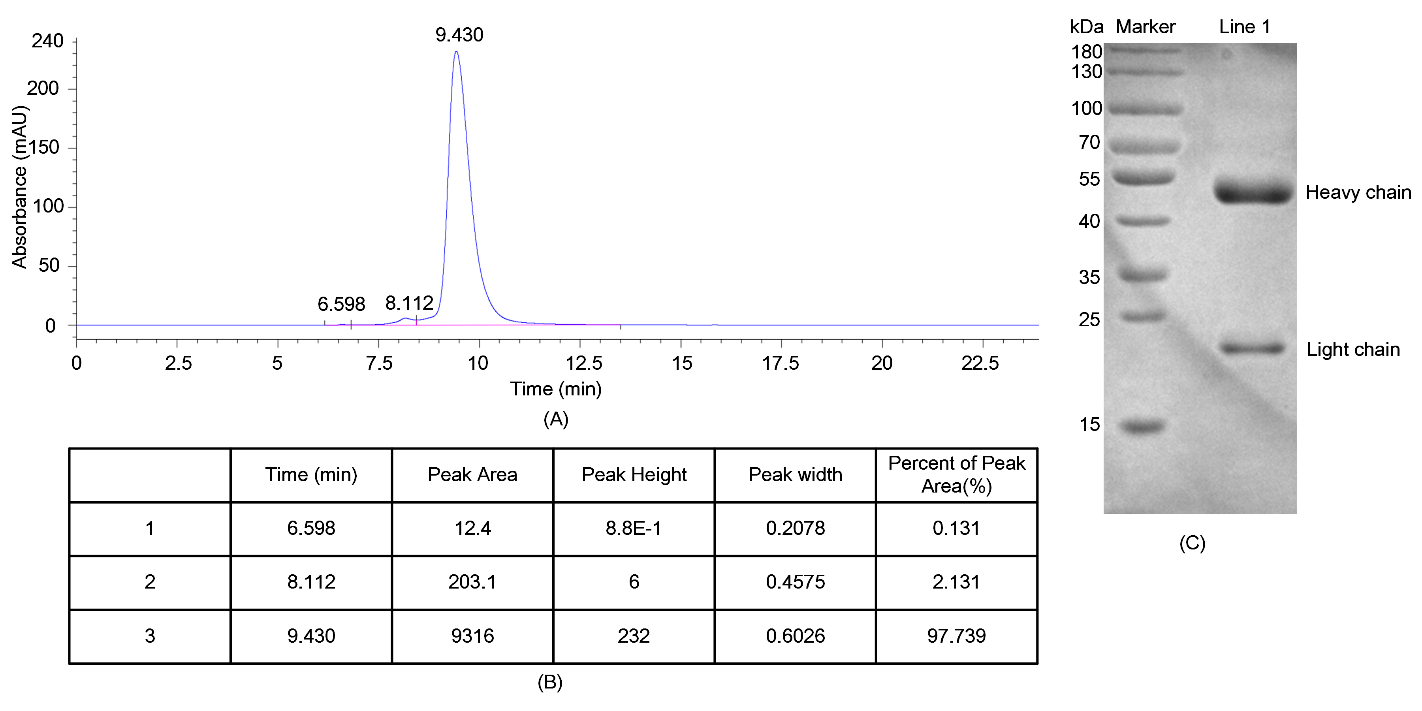
**

Supplement: S7 Fig — Size exclusion chromatography (SEC) and (SDS-PAGE) assays have been performed to determine the purity of antibody 4D3. (A) A peak indicating the existence of antibody 4D3 was detected at 9.43 min. (B) the purity of antibody 4D3 is 97.7%. The parameters of SEC are: Flow rate: 0.7ml/min; Temperature: 25°C; 40 μL antibody 4D3 is injected into the column which is purchased from Aglilent Technologies (Agilent AdvanceBio SEC). (C) 4D3 was separated with a reduced SDS-PAGE, and two clear bands indicates heavy chain (50kDa) and light chain (20kDa). No irrelevant band was observed. (DOCX) [file pone.0192506.s007.docx]

**S8 Fig**

**
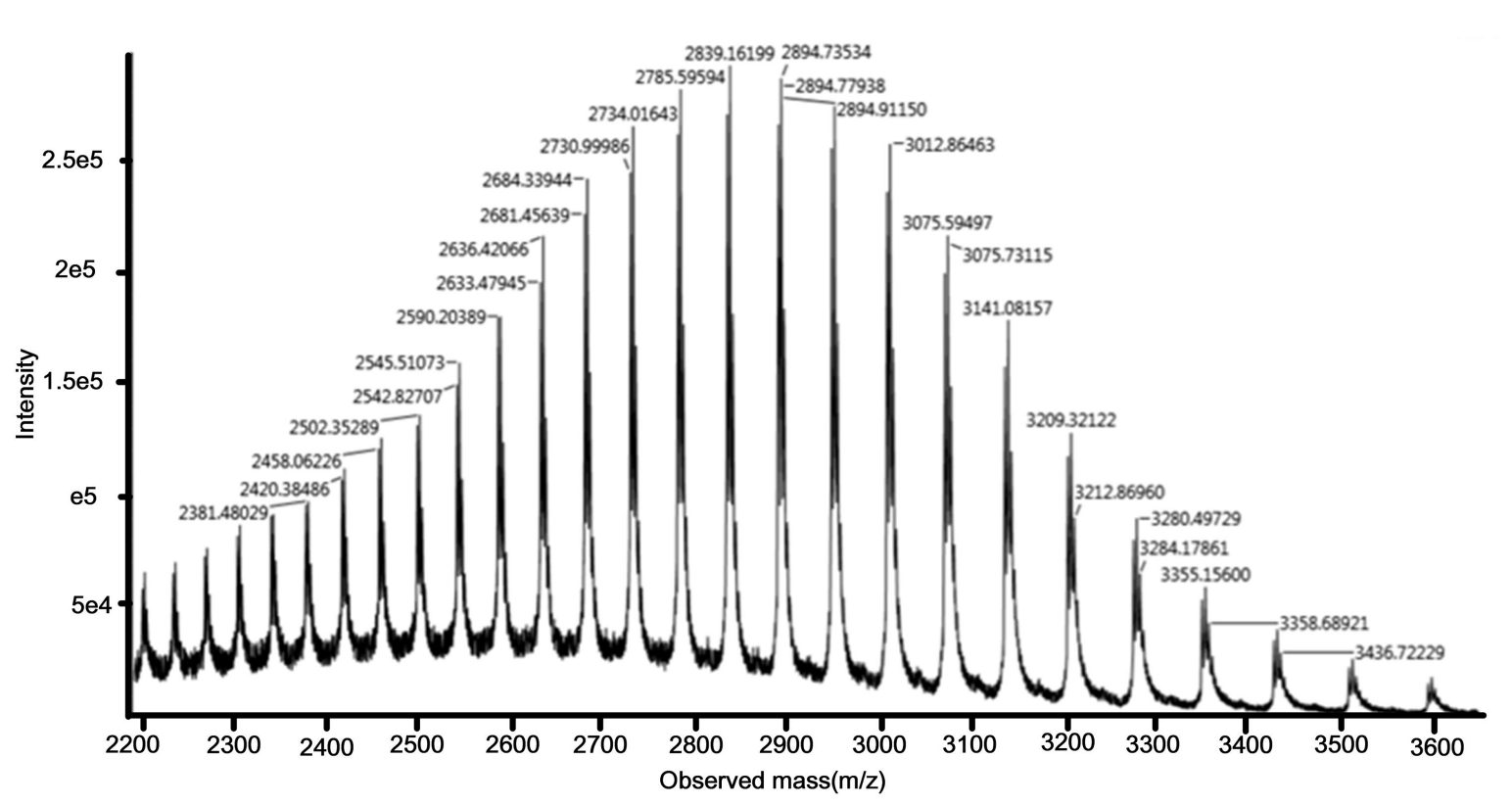
**

Supplement: S8 Fig — The accurate molecular weight of 4D3 antibody was analyzed by LC-MS. The result exhibits 27 clear and independent peaks, without any irrelevant noise signal, demonstrating that monoclonal antibody is acquired. (DOCX) [file pone.0192506.s008.docx]

**S9 Fig**


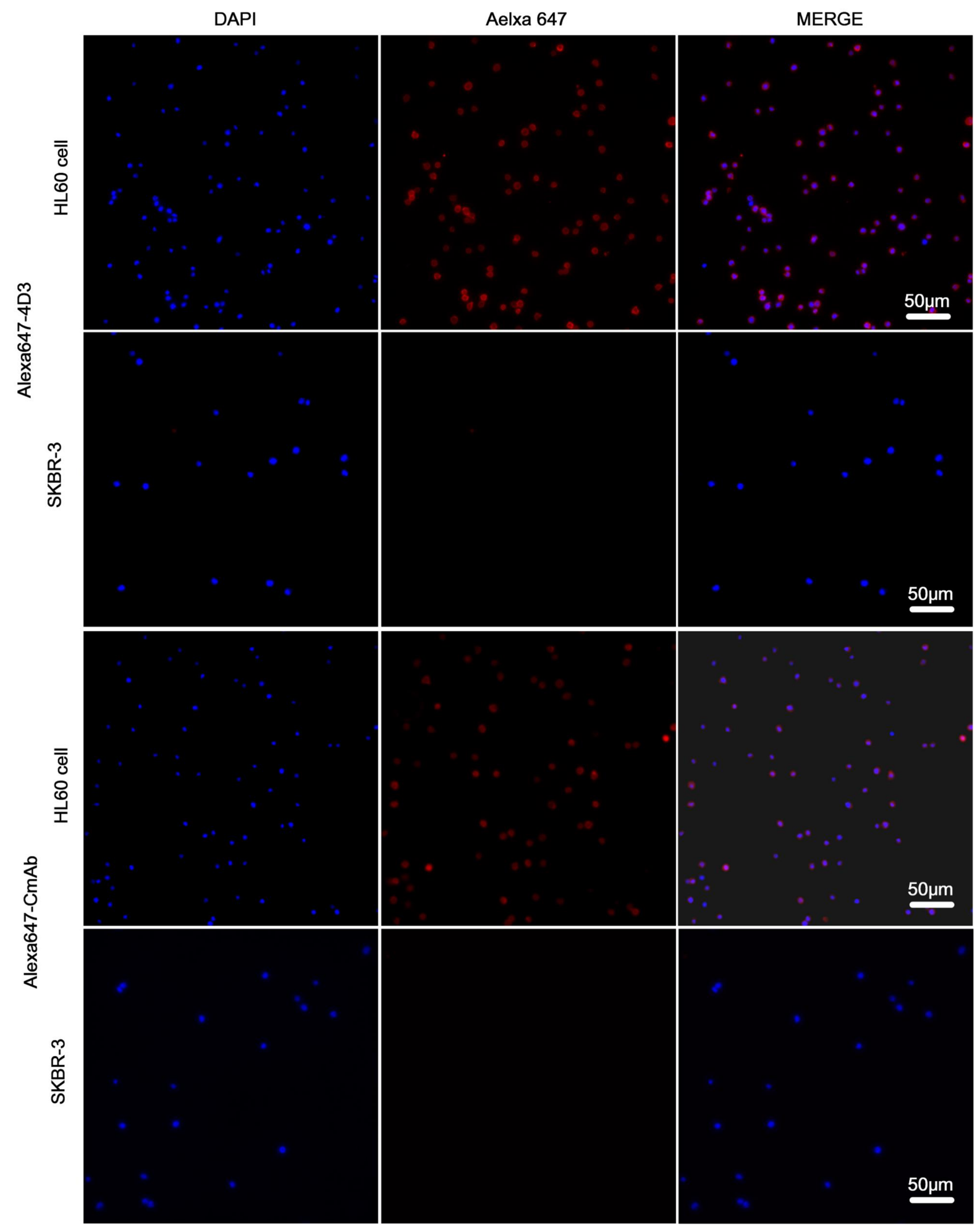

Supplement: S9 Fig — To evaluate the specificities of Alexa647-4D3 and Alexa647-CmAb, SKBR3 and HL60 cells were stained by Alexa647-4D3 and Alexa647-CmAb, respectively. In this figure, blue spots represent cell nucleic stained by DAPI. As expected, neither Alexa647-4D3 nor Alexa647-CmAb bound to SKBR3 cells. As to the binding with HL60 cells, Alexa647-4D3 shows similar or slightly better performance with Alexa647-CmAb. Under the same optical conditional, Alexa647-4D3 is with higher fluorescent intensity than Alexa647-CmAb. Scale bar:50 μm. (DOCX) [file pone.0192506.s009.docx]

**S10 Fig**

**
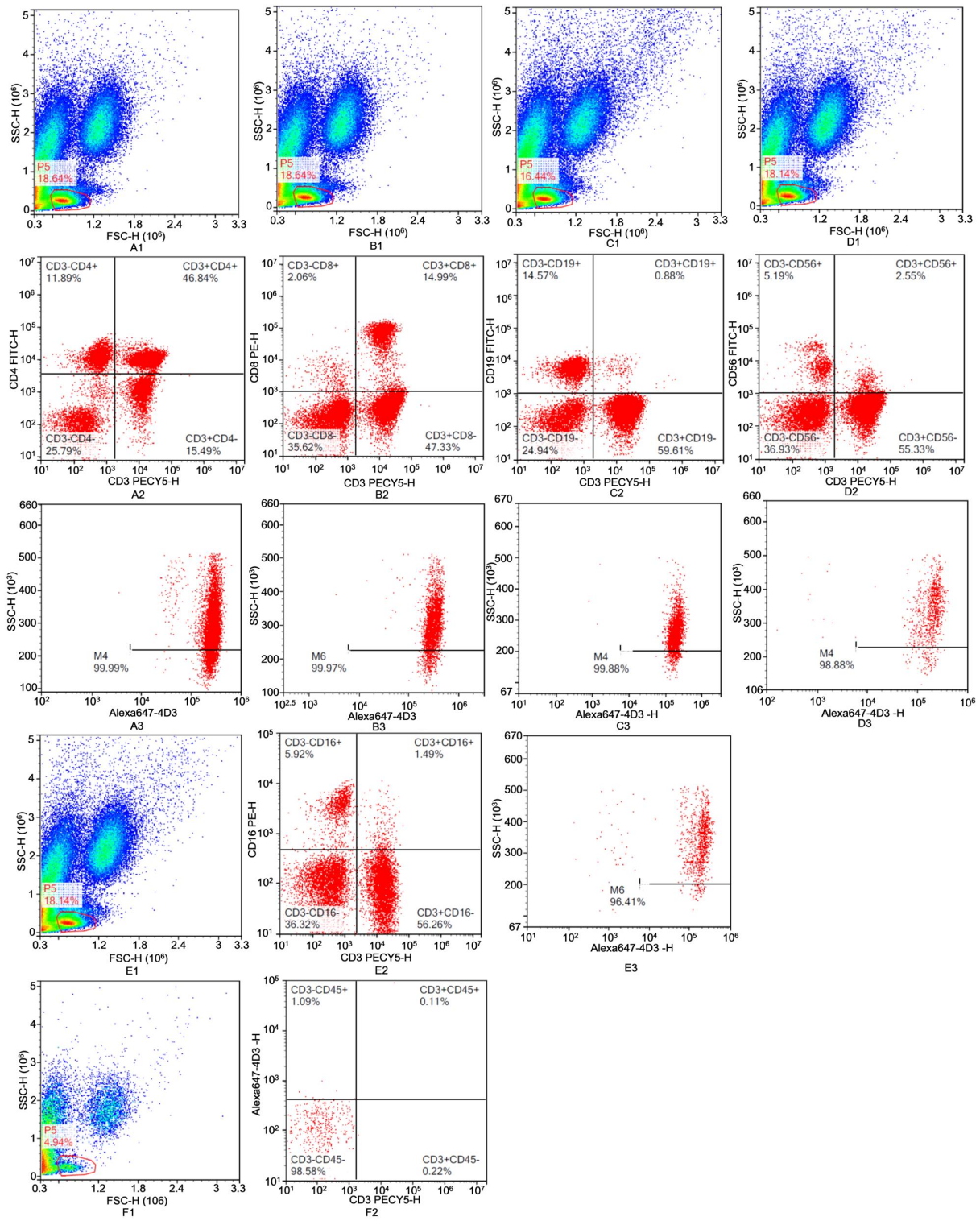
**

Supplement: S10 Fig — Flow cytometry was used to evaluate the binding between Alexa647-4D3 and lymphocyte subsets. CD3 (PECY5 labelled), CD4 (FITC labelled), CD8 (PE labelled), CD16 (PE labelled), CD19 (FITC labelled), and CD56 (FITC labelled) antibodies were introduced to differentiate lymphocyte subsets. Alexa647-4D3 was used to label all lymphocytes. S10A1, S10A2, S10B1 and S10B2 Fig demonstrated that T cells (CD3+CD4+ and CD3+CD8+) accounted for 61.83% of total lymphocyte amount while S10A3 and S10B3 Fig revealed that about 99.9% of T cells exhibited obvious binding with Alexa647-4D3. Similarly, B cells (CD3-CD19+) accounted for 14.57% of total lymphocyte amount (S10C1 and S10C2 Fig) while about 99.9% of B cells exhibited obvious binding with Alexa647-4D3 (S10C3 Fig); NK cells (CD3-CD56+CD16+) accounted for 5.1% of total lymphocyte amount (S10D1, S10D2, S10E1 and S10E2 Fig) while more than 96.4% of NK cells exhibited obvious binding with Alexa647-4D3 (S10D3 and S10E3 Fig). S10F1 and S10F2 Fig were negative controls in which no antibody was used. (DOCX) [file pone.0192506.s010.docx]

**S11 Fig**

**
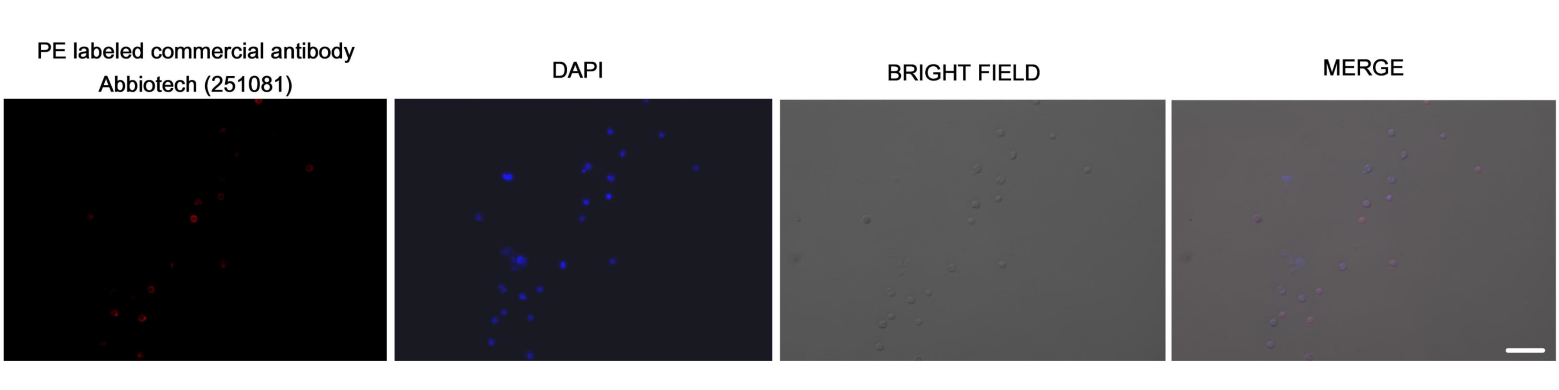
**

Supplement: S11 Fig — Before applying commercial mAbs on CTC identification, we tested the binding between commercial mAbs and HL60 cells. One of them (Sino Biological 10086-MM05-F-25) results in no binding at all, while another one (Abbiotec 251081) is with limited binding efficiencies. (Only about 60% of HL60 cells was recognized, as shown in the S11 Fig). The binding between PE-labelled Abbiotec 251081 antibody and HL60 cells shows that about 60% HL60 cells were recognized. Scale bar: 20 μm. (DOCX) [file pone.0192506.s011.docx]
